# “Time for Recovery” or “Utter Uncertainty”? The Postponement of the Tokyo 2020 Olympic Games Through the Eyes of Olympic Athletes and Coaches. A Qualitative Study

**DOI:** 10.3389/fpsyg.2020.610856

**Published:** 2020-12-22

**Authors:** Violetta Oblinger-Peters, Björn Krenn

**Affiliations:** ^1^Department of Sport Science, Martin Luther University of Halle-Wittenberg, Halle, Germany; ^2^Department of Sports Sciences, University of Vienna, Vienna, Austria

**Keywords:** Olympic Games Tokyo 2020, postponement, content analysis, COVID-19 pandemic, coping, athletes, coaches

## Abstract

The current COVID-19 pandemic has affected the entire globe, including the world of high-performance sports. Accordingly, it has been widely assumed that the thereby caused postponement of the Tokyo 2020 Olympic Games could have negative psychological impacts for aspirants, since they were halted abruptly in the pursuit of their Olympic endeavors and their daily lives drastically altered. Considering the sudden nature of the pandemic, few researchers, if any, have yet scrutinized the individual experience of Olympic aspirants. This qualitative study examines the subjective perceptions of the Tokyo 2020 Olympic Games postponement among Austrian Olympic athletes and coaches. To this end, 21 Austrian athletes (13 male, 8 female; mean age = 26.67 ± 4.93 years) and six male coaches were recruited through a criterion-based purposive sampling strategy. Five athletes had already qualified for the Olympic Games in Tokyo 2020 and 15 athletes were still in an ongoing qualification process. Data was collected by means of short written statements, elicited via open-format questions on an anonymous online survey platform. In order to infer meaning from the text, a qualitative content analysis with an interpretative focus was conducted inductively, which allowed for deriving alternative explanations of findings. The results support the notion that the Olympic postponement was experienced in myriad ways by affected participants. Three general themes comprised of several meaning units of different levels of abstraction were created from the text data. Many respondents experienced an immediate emotional reaction to the postponement characterized by confusion, disappointment and/or relief. Participants associated multiple consequences with the postponement, such as the prolongation of physical and psychological pressure, a lack of motivation, concerns about future performance, living and their occupational career, but also the opportunity for performance improvement and recovery. Respondents displayed various coping strategies, such as distancing themselves from sports, cognitive reframing, appealing for acceptance, and planning behavior. This study gleans first insights into the idiosyncratic experience of the Olympic Games 2020 postponement among Austrian aspirants. The findings could serve to assist sport psychologists in their applied practice by informing them about athletes’ and coaches’ needs in their Olympic preparation during the ongoing pandemic.

## Introduction

When the Coronavirus forced the World Health Organization to declare a public health emergency of international concern and categorize the disease as a global pandemic on 11th March 2020 (WHO, 2020), the world of sport began to wait for a decision on behalf of the International Olympic Committee (IOC) about the Olympic Games’ commencement in Tokyo in July 2020. The IOC became increasingly criticized for reacting hesitantly to the dramatically evolving pandemic by the international sport community and health experts alike. With complaints growing louder and several national Olympic committees taking a clear stand by withdrawing from the Games, the unthinkable eventually happened. On 24th March the IOC and Japan’s Prime Minister, Shinzo Abe announced that the Tokyo 2020 Olympic Games would be postponed until 2021 (Rich et al., 2020). It was the first time since the opening of the modern Olympic Games in 1896 that the world’s most prominent sport event would be delayed in peace time, a fact that highlights the magnitude of the unprecedented health crisis caused by the coronavirus outbreak for the world of sport (Taku and Arai, 2020).

The COVID-19 pandemic and the subsequent postponement of the Tokyo 2020 Olympic Games raised broad discussions within the world of sport about the challenges Olympic athletes and coaches are encountering. The issues ranged from handling social distancing, social isolation, and loss of social support to dealing with career disruption, uncertainties about the Olympic qualification status and process, limitations and bans of training possibilities as well as insecurities about future funding, sponsorship contracts and individuals’ health status (Schinke et al., 2020a,b; Toresdahl and Asif, 2020). From a holistic developmental perspective (Wylleman, 2019), the COVID-19 pandemic can be regarded as a significant career development barrier for athletes, bringing about changes in their athletic, psychological, academic-vocational, financial and legal development (Stambulova et al., 2020). On top of these unique demands produced by the current pandemic, the Olympic Games are considered a career change-event (Samuel et al., 2016), comprised of a multi-phase transition process (Stambulova et al., 2012; Wylleman et al., 2012), and entailing as such very specific challenges for athletes. Against this backdrop, the authors of this study were curious how Olympic aspirants would experience the unprecedented postponement.

In numerous research projects world-wide, increased levels of stress, anxiety, and depressive symptoms as well as feelings of anger, grief, fear, confusion, and emotional exhaustion among the general population were documented as negative psychological effects of the COVID-19 pandemic and national governments’ actions to restrict the spread of the virus and limit its detrimental impacts (Ahmed et al., 2020; Brooks et al., 2020; Chakraborty, 2020; Mazza et al., 2020; Qiu et al., 2020; Rajkumar, 2020; Wang et al., 2020). Moreover, studies carried out among elite athletes also reported a rise in stress levels, depression and anxiety symptoms due to the current pandemic (di Fronso et al., 2020; FIFPRO, 2020). It could be assumed that in Olympic sport particularly, people’s mental health and well-being could be negatively affected. In their pursuits toward excellence, the challenge of facing a global health crisis combined with seeing the Olympic Games delayed (presumably a symbol of their visions, lifelong dreams, or for the culmination of their professional strivings and ambitions), could put Olympic aspirants at greater risk of experiencing adverse psychological effects. Concerns were voiced that repercussions of the pandemic would threaten the mental health of Olympic participants whose daily routines were disrupted from one moment to the next as competitive seasons had come to an abrupt halt and training facilities around the world were closed (Schinke et al., 2020a; Toresdahl and Asif, 2020).

The announcement of the official IOC decision sparked an abundance of interviews and global media coverage of future participants’ reactions to the postponement. Personal social media feeds of Olympic aspirants testified to the emotional turmoil that the events had triggered within the world of Olympic sports. In addition, elevated demands for psychological online support through sport psychologists were reported following the outbreak of COVID-19 and the implemented confinement measures (Hossien Mehrsafar et al., 2020). Taken together, the question was raised whether Olympic candidates possess sufficient and adequate coping resources to deal adaptively with the unprecedented situation (Hossien Mehrsafar et al., 2020). Elite athletes have been shown to employ different coping strategies to meet the demands imposed on them in the pressurized environment of high-performance sport (Nicholls and Polman, 2007; Nicholls, 2010a). Considering the societal consequences of the COVID-19 pandemic and the postponement of the Olympic Games in Tokyo 2020, it seems open if and how athletes and coaches employ different coping strategies. Extant literature differentiates between problem-focused coping (e.g., actions of problem solving, planning, or information seeking), emotion-focused coping (e.g., seeking social support, relaxation techniques, or acceptance), meaning-focused coping (e.g., searching for meaning in adversity, drawing on values, beliefs and goals, or making causal attributions), and avoidance coping, comprising behavioral (e.g., removing oneself from the stress-inducing situation) as well as cognitive (e.g., blocking thoughts or cognitive distancing) efforts to withdraw from a stressful situation (Krohne, 1993; Lazarus, 1999; Folkman and Moskowitz, 2004; Nicholls and Polman, 2007; Polman, 2011; Guo et al., 2013). It seemed likely to find Olympic aspirants resort to various coping strategies in response to the postponement of the Olympic Games.

However, due to the dynamic progression of the health crisis, there is a lack of empirical work within the field of sport psychology on how this unique population actually experience the Olympic postponement. Assumptions about which feelings, concerns, frustrations, fears or reliefs accompany the IOC’s decision on the part of affected aspirants, and which coping strategies they might resort to in order to overcome encompassed difficulties, are thus far of hypothetical nature only. Currently, few studies examining individual perceptions of the ongoing COVID-19 crisis among Olympic athletes and coaches have been published (e.g., Clemente-Suárez et al., 2020; Taku and Arai, 2020). With the world scrambling up to cope with the implications of the current health crisis, a detailed exploration of perceived challenges and associated coping responses as well as additional idiosyncrasies through participants’ lived experience of the delay of the Olympic Games is still missing. Designed to address this gap in knowledge, the purpose of the present paper is to provide subjective interpretations of this unprecedented situation through the eyes of Olympic aspirants. Hence, a qualitative approach to inquiry was used to afford a deeper insight into this novel area of interest (Patton, 2002), centralizing two research questions: (1) How do Olympic athletes and coaches experience the postponement of the 2020 Tokyo Olympic Games? (2) What consequences do they expect?

## Materials and Methods

### Philosophical Positioning

The authors of this study endorse the idea that people’s realities are shaped through their individual and subjective perceptions and thereby commit to epistemological constructionism (i.e., knowledge is constructed and subjective) and ontological relativism (i.e., multiple created, mind-dependent realities exist; Ponterotto, 2005; Smith and McGannon, 2017). Accordingly, the goal of the study was to acknowledge and show that the situation of seeing the Olympics Games delayed due to an unforeseen global health crisis can be experienced in myriad ways by different individuals and that it impossible to know what this unique situation means and entails for them.

### Participants

Participants were recruited through a criterion-based purposive sampling strategy (Sparkes and Smith, 2014) to ensure that they shared predefined inclusion criteria characteristics. Accordingly, 78 Austrian athletes and 16 Austrian coaches in total were invited via email or private message on Facebook to participate in the study. These athletes were selected due to: (a) successfully completed qualification for the Olympic Games in Tokyo 2020 or (b) high probability of qualification for the Olympic Games in Tokyo 2020. The coaches were corresponding coaches of the aforementioned athletes whose German language skills were deemed sufficient to provide a detailed written description of their lived experience. Twenty-one athletes (13 male, 8 female; mean age = 26.67 ± 4.93 years) and six male coaches (mean age = 46.67 ± 9.73 years) followed the invitation and participated in the study. Five of these athletes stated that they were already qualified for the Olympic Games in Tokyo 2020, 15 athletes stated that they were in an ongoing qualification process and one athlete did not answer this question. Eight athletes had participated in previous Olympic Games, whereas 13 athletes stated trying out for their Olympic debut. Concerning their sports: Eighteen athletes and five coaches categorized their sport as individual sports, while three athletes and one coach categorized their sport as team sports. The study took place from 21st April to 19th July 2020. Data collection was halted on the grounds that the lockdown phase of the COVID-19 pandemic in Austria was over and the contextual circumstances had vastly changed for additional potential participants. Out of the 27 respondents, 24 took part in the study within the first 9 weeks. Three participants responded within the last 3 weeks of the study.

### Procedure and Data Collection

Potential study participants were contacted via social media and email and provided with an online link to a survey platform, which could be accessed in a computer or mobile version. The research was introduced as a study to explore how Austrian Olympic athletes and coaches personally experience the postponement of the Olympic Games caused by the Coronavirus pandemic. In the introductory text, special care was attributed not to influence participants in order to capture their idiosyncratic views. Before directing participants to the question, the procedure was described and the athlete or coach was informed of his or her right to withdraw from the study at any time. After this, participants were asked for their explicit consent. It was made clear that the researchers would not be able to infer personal identities from the data and that the study intended to provide an anonymous setting for participants to share their subjective experience in a written statement. The open-format questions that participants were asked to answer read as follows: “Please describe what the postponement of the summer Olympic Games of Tokyo 2020 means from your perspective. What does this decision entail for you personally?”

The method of prompting written statements from participants stems from research conducted by Hunger and Böhlke (2017). These authors developed the method of a “short written narration” (p.5) further by asking study participants to elicit a statement about a specific situation that was subjectively perceived as boundary overstepping in the context of physical education. The method of prompting written statements in an online format has so far received little attention in methodological reflections in the context of qualitative research (Schiek, 2014). Despite some drawbacks of this method (e.g., participants’ varying ability to express internal experiences in writing and the invisibility of participants’ omissions and corrections of content for the study authors; cf. Bampton and Cowton, 2002; Schiek, 2014), the rationale behind applying this method of data collection in the current study rests on several grounds. Firstly, the online link was available around the clock from anywhere so that study participants could access the survey at their convenience, meaning whenever they felt ready to self-disclose. In this sense, the authors intended to offer an anonymous setting for participants, in which they could reflect freely on their private experiences, at an autonomously chosen point in time, and what is more, without being influenced by the study authors. This aspect was vital for the study design, which intended to allow participants to express personal opinions and views that they might be hesitant to disclose in a public media interview or in an online interview conducted face-to-face with the study authors. Since both authors are currently providing sport psychological services to athletes and coaches in Austria, they were potentially personally known by participants. Furthermore, the authors chose the data collection method in order to help participants process their experiences and feelings. Since writing involves arranging, revising and structuring content, it allows experiences to be processed directly (cf. Schiek, 2014).

Following the writing prompt, participants were asked to provide information regarding their age, gender, actual qualification status, previous participation in Olympic Games, the nature of their sport, and whether they were athlete or coach. No additional biographical or sport-related details were obtained from participants due to the aforementioned concerns about ensuring their anonymity. The statements, which respondents produced, were on average comprised of seven lines, ranging from two to 37 lines between individuals.

### Reflexivity

In order to increase the credibility of their claims, the authors acknowledge their own standpoints and feel the responsibility to clarify their positionality in regard to the topic and the population under investigation (Levitt et al., 2018). The first author recognizes her complex positioning as a former and long-term member of the Austrian Olympic Team, for which she competed in three Olympic Games and won one individual medal. After retiring from her athletic career, she started to work as a certified mental performance consultant in Austria. As such, she is currently supporting clients in their preparation for the next Olympic Games. The second author has been in charge of the Austrian Team at Olympic Games as sport and exercise psychologist for several Olympic cycles. He has extensive experience in providing support for athletes and coaches in high performance settings and additionally holds a post doc position at the University of Vienna in the field of sport psychology. Against this backdrop, the authors are aware that they were able to make use of privileged communication pathways to establish contact with potential study participants. Furthermore, it is worth noting that anonymity and the impossibility to link data to individual replies seemed to be questioned as some participants emailed the authors asking them if further information was needed from them. Since the high-performance sport community in Austria is rather small, athletes, coaches, stakeholders and sport psychological support staff know each other personally in most cases, which added to the challenge of establishing confidence and trust among the study participants that their anonymity would be safeguarded at all times.

### Data Analyses

The first and second author individually performed a qualitative content analysis with the goal of gathering knowledge and enhancing understanding of the phenomenon under study (Downe-Wamboldt, 1992). In line with preliminary reflections about the research design, this analysis method was conducted with an interpretative focus while systematically classifying and identifying patterns of meaning. Following the differentiation of Hsieh and Shannon (2005), the authors employed a conventional approach to content analysis. This type of content analysis was aligned with the study’s purpose to develop a deeper understanding of a recent phenomenon, for which research literature is lacking. Without imposing preconceived categories or theoretical considerations, a conventional content analysis is data-driven, and thereby derives knowledge from participants idiosyncratic perspectives (Hsieh and Shannon, 2005).

In a first step, the authors familiarized themselves with the data by reading the entirety of the texts repeatedly to get a sense of the whole (Tesch, 1990). Next, the authors analyzed the text inductively to allow themes and categories to appear from the data (Patton, 2002; Mayring, 2015). Meaning units, defined as “a segment of text that is comprehensible by itself and contains one idea, episode, or piece of information” (Tesch, 1990, p. 116), were identified and labeled as codes. In this article, codes are understood as condensed meaning units and as such employed as “tools to think with” (Coffey and Atkinson, 1996, p. 32), intended to look at the data in new ways. The labels were mostly based on the wording of the text and contained key words (Hsieh and Shannon, 2005). In a next step, the codes were grouped into subcategories and categories representing higher levels of abstraction. A category can be considered as a thread throughout the codes (Graneheim and Lundman, 2004). As a final step in the independently performed analysis, recurring meanings across categories were inferred and labeled as themes (Polit and Hungler, 1999). A theme can also be thought of as a thread of an underlying meaning, but on an even higher level of abstraction, since it encompasses latent content of the text, obtained through interpretative analysis. The interpretative nature of themes makes it possible for codes and categories to be sorted into more than one theme, as themes are not necessarily mutually exclusive (Graneheim and Lundman, 2004).

Once the analysis was conducted by the two authors individually, the researchers met on several occasions to present their thematic framework to each other and to discuss their interpretations of the underlying meaning (i.e., the latent content) of the text. Despite the content analysis being depicted as a linear process, it is important to note that the derivation of meaning from a text is performed in a rather circular manner by moving back and forth between the text as a whole and its segments. As described earlier, the research design was underpinned by constructivist epistemological beliefs and a commitment to a relativist ontology. In line with these beliefs, the authors assume that it is impossible to produce theory-free knowledge as reality can be interpreted in a variety of ways. This in turn, implies the idea that understanding depends on subjective interpretation. Consequently, the authors decided against the use of the popular method of member checking as a form of quality control and with the aim to ensure rigor (cf. Smith and McGannon, 2017). Likewise, interrater reliability was regarded as irrelevant to this study, since a quest for consensus among researchers as Kvale (1996) put it can “lead to a tyranny by the lowest denominator: That an interpretation is only reliable when it can be followed by everyone, a criterion that could lead to a trivialization of the interpretations” (p. 181; Smith and McGannon, 2017). Instead, the authors chose to first perform the content analysis independently and discuss their results afterward without aiming for interpretative agreement. In this regard, they factually acted as critical friends to each other, promoting reflexivity by challenging each other’s views and their respective construction of knowledge (Cowan and Taylor, 2016; Smith and McGannon, 2017). In the methodological debate about strategies to achieve rigor in qualitative research within sport and exercise psychology, the critical friend procedure is gaining momentum (e.g., Gustafsson et al., 2008; Hill and Hemmings, 2015) and was chosen by the authors in order to increase the quality and trustworthiness of this interpretivist study.

## Results and Discussion

Since description and interpretation of the underlying meaning are closely related in this qualitative approach, the “Results and Discussion” sections are combined in order to avoid redundancy. Three central themes related to the perception of the postponement of the Tokyo 2020 Olympic Games were inferred from the data: (1) Immediate emotional reactions, (2) Perceived consequences of the postponement, and (3) Coping with the situation. The detailed structure and composition of the derived categories, subcategories and codes are presented in Tables 1–3.

**TABLE 1:** Hierarchical structure of the theme “Immediate emotional reactions”.

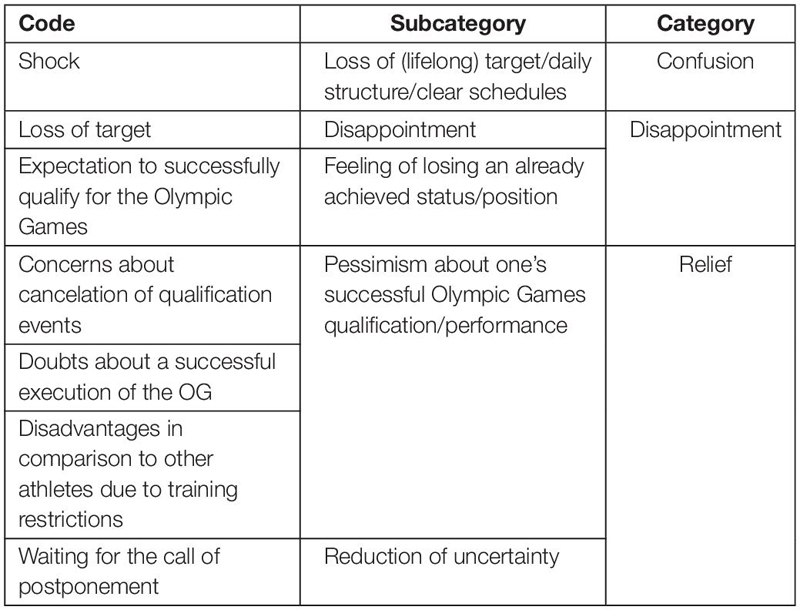

**TABLE 2:** Hierarchical structure of the theme “Perceived consequences of the postponement”.

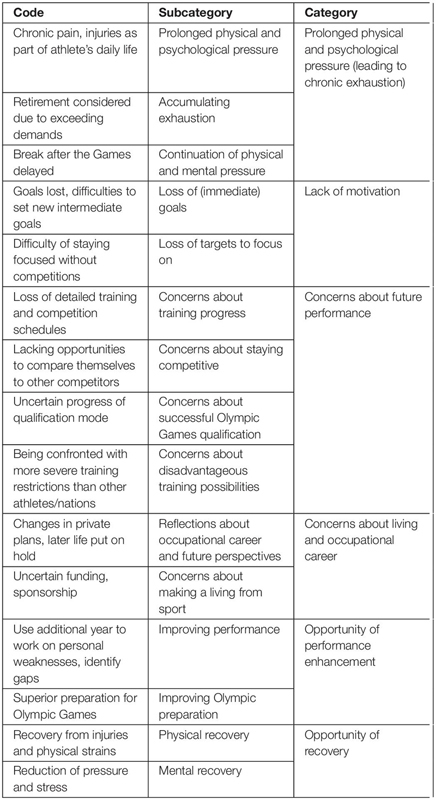

**TABLE 3:** Hierarchical structure of the theme “Coping with the situation”.

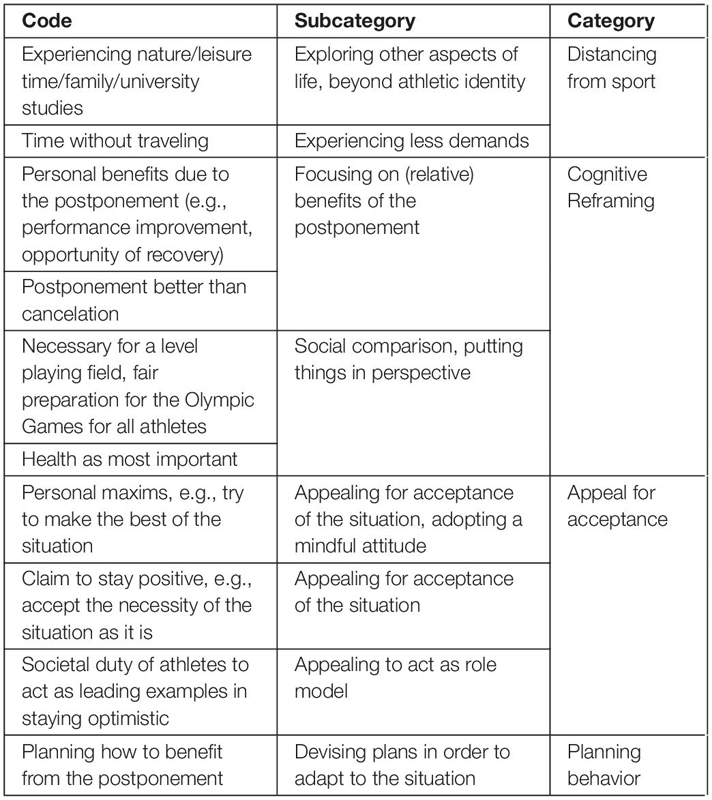

### Immediate Emotional Reactions

Table 1 illustrates the codes, subcategories, and categories, which were created from participants’ texts relating to their immediate emotional reactions. The categories and main emotional states ascribed were confusion, disappointment, and relief.

#### Confusion

In many cases, participants described the way they reacted to the announcement of the postponement in distinguishable phases. Thus, they often recalled their initial reaction as a sense of shock and confusion. As this respondent put it: “Of course I was shocked at first and for a short time I didn’t know what to think about it or how to deal with the situation at all” (id13). Another participant asserted: “For me personally, it was the only right decision, even if it was a small shock at first” (id23). Several participants associated this shock moment with a feeling of uncertainty. Often, the underlying factors of having no exact dates and schedules to structure their yearly training accordingly, in combination with being left in the dark about possible changes to the ongoing Olympic selection process and rules, were described as challenging. One athlete stated: “As a consequence, all athletes find themselves in utter uncertainty. I would describe that as the most difficult issue” (id4). This finding was somehow expected as it reflects the general assumption within the field of sport and exercise psychology that elite athletes, in terms of being high-performance career professionals, evolve based on structured short- and long-term plans, which logically permit outcomes such as improved and consistent performance and viability into the future (Schinke et al., 2020b).

#### Disappointment

Feelings of disappointment and regret were frequently reported as a first emotional reaction: “Immediately after the announcement of the decision, it was a challenge for me to process everything, because I had invested a lot in the upcoming selection and had already completed a big part of it successfully!” (id12). One coach explained how the Games’ postponement was experienced as tragic, because it stopped the team’s ongoing Olympic selection at its final stage: “Also, we were really close to qualifying, there were only 2 competitions left. Now everything is “up in the air” again and there is a certain insecurity” (id14). There is a growing awareness within the field of sport psychology that even in a “normative” Olympic cycle, athletes are prone to disappointment in their Olympic endeavor, which might impact their well-being and ultimately their mental health negatively (Henriksen et al., 2020). With this in mind, the extended preparation phase caused by the postponement understandably represents an important challenge for Olympic aspirants.

#### Relief

Since the phase proceeding the IOC’s announcement was characterized by an agonizing uncertainty about scheduled events and plans in general, the official call served to reduce this uncertainty and was consequently experienced as relief at first. Some participants felt relieved that difficult recent training conditions were now judged as less unfavorable: “It is a relief, because we were not able to train in the last month” (id5). Another athlete added: “Following a stage of uncertainty, the postponement of the Olympic Games to 2021 felt like a big relief to me. Moreover, training conditions were very different from country to country, the postponement of the Games allows for a fair preparation” (id17). Numerous respondents had pessimistic views about the continuation of their qualification process and feared negative changes to their selection mode before the delay of the Olympic Games was formally announced. Therefore, they felt relieved about the formal decision of the IOC, as it ended speculation about the cancelation of upcoming qualification events. This athlete stated: “Personally, I have to say that the postponement was a relief. I wasn’t qualified 100% yet and was scared that because of the corona crisis there would be no more competitions, so that there would be no more chances to meet the selection criteria for me. My big goal was always the Olympics, and not being able to participate because of something like this, made me think a lot” (id27).

These comments exemplify that Olympic athletes and coaches lived through diverse concerns that COVID-19 will affect their actual situation, and even more importantly, their future perspective negatively. As already shown in other population groups besides elite athletes and coaches, increased levels of stress, anxiety and depression were documented as a psychological consequence of COVID-19 (Ahmed et al., 2020; Brooks et al., 2020; Chakraborty, 2020; Mazza et al., 2020; Qiu et al., 2020; Rajkumar, 2020; Wang et al., 2020). The data of this study might be in line with these quantitative findings. As such, the reported mixture of feelings of shock, confusion, disappointment and relief might represent a typical progression. Accordingly, the decision of the postponement sparks a moment of shock at first, as the major target of athletes and coaches is propelled into the future. As this shift of time perspective has a major impact on short-term as well as on long-term plans of Olympic aspirants, a phase of confusion might follow, in which they have to factually process the novel situation and re-orientate themselves in order to adapt. By realizing the situation and assessing its individual, multidimensional consequences, feelings of disappointment, but also relief might ensue depending on individually rated positive and negative implications of the postponement and on one’s perceived ability to manage stress. The depicted progression bears resemblance to Kübler-Ross (1969) model of grief, which comprises five (non-linear) stages as reactions to loss i.e., denial, anger, bargaining, depression, and acceptance.

### Perceived Consequences of the Postponement

Table 2 shows the hierarchical structure of the codes, subcategories and categories that were identified from the participants’ answers relating to the perceived consequences of the postponement of the Tokyo 2020 Olympic Games. The identified topics were grouped into six categories.

#### Prolonged Physical and Psychological Pressure

Numerous participants expected the Olympic postponement to bring about negative consequences. A recurring issue in the data was the notion of an extended period of mental and physical pressure due to the delayed Games. Multiple athletes and coaches mentioned chronic pain, injuries, and physical and emotional stress resulting in exhaustion as characteristics of the daily life of professional athletes: “During the entire selection phase (selection had been going on for 2 years already), I had, among other things, to constantly deal with injuries. As a high-performance athlete, among other things, you are forced to permanently exceed your limits and often you don’t give your body the time it would actually need” (id4). In some cases, this even led to the contemplation of an early career termination, since the demands of prolonged physical and emotional hardship (by at least another year) seemed too overwhelming: “For me personally, this postponement represents a challenge. I am often injured and have been in chronic pain for years already. The plan was to try as good as possible to give it 100% until the Games and then to slowly end the career. To train a whole year longer for the Olympics is going to be a challenge for me, mentally and physically. After the announcement that the Games will be postponed, I was wondering for a long time if I should continue at all” (id12). The topic of career ending was also mentioned by one coach, who confessed that he had very different plans for his life since he was planning to retire after the Games, but now has to “add another year” (id14). The toll that the long-lasting and draining Olympic qualification phase takes on athletes was paramount. As such, it was noticeable in the words of this coach, describing how much effort had been put into the preparation since 2016 and concluding that “the athletes were quite worn out already” (id14). In one athlete’s statement the unwanted interruption and stretch of time was especially pronounced: “One more year until the event that we have been preparing for 10 years will finally take place” (id19).

These depictions indicate a high level of mental and physical exhaustion, which might represent a core dimension of burnout (Maslach et al., 2001; Schaufeli and Buunk, 2003; Gustafsson et al., 2016) comprising both physical and emotional aspects (Gould et al., 1996; Cresswell and Eklund, 2006). Informed by this knowledge, the statements could be interpreted as (early) signs of bodily and psychological fatigue due to demands of the pressurized environment of elite sport. It is known today that the specific demands of elite sport such as high training loads, abundance of calendared competitions, inflexible training schedules and mental pressure can act as potential stressors to athletes and even threaten their mental health (Rice et al., 2016; Küttel and Larsen, 2019). Consequently, it is plausible to assume that athletes and coaches who expressed their fatigue in this study are even more at risk of poor mental health now as stress and negative emotions caused by the pandemic are added to the mix.

#### Lack of Motivation

The feeling of an accumulating exhaustion was directly linked to a motivational low by some participants, who attributed this to the fact that the outlook on a break after the Olympic Games was deferred for one more year: “As I was expecting at least a longer break after the Olympic Games, the postponement turned a lot of things upside down. Because I was really looking forward to this break, it is actually very difficult to maintain the motivation at the moment” (id24). Another aspect affecting athletes and coaches’ motivation negatively, became apparent in previous statements already: The loss of calendared competitions and thereby clear targets to peak for. As one athlete stated “in a year without any major event, it seems challenging to keep the focus, as the main target is far away and there are no conducive intermediate goals” (id33). One participant reflected: “In the first moment after the final decision of the postponement for me personally, it was not as easy as expected. The questions of how to set new goals and what I might have to adjust in my private life turned out difficult for me at the beginning” (id37). Another athlete talked about how the postponement was a shock “because you train hard all winter long to qualify and suddenly this goal is snatched away from you” (id54). These statements elucidate the impact that the postponement of the Olympic Games has on athletes and coaches’ goal setting, an aspect previously emphasized by Schinke et al. (2020a, b). According to the vast psychological literature and pertinent studies, goal setting is relevant for effective goal striving and can ultimately lead to enhanced performance (Locke and Latham, 2002), which is why it is one of the most widely used interventions by practitioners (Weinberg and Butt, 2011; Healy et al., 2018). By setting oneself goals that are aligned with one’s personal values and pursuing these autonomously, athletes see purpose and meaning in sport and life in general. In this sense, setting oneself meaningful and personally significant goals was identified as a protective factor against mental illness and can in turn serve to increase mental health in the high-performance sport context (Mousavi et al., 2017; Küttel and Larsen, 2019). Thus, effective goal setting might help athletes and coaches in a healthy pursuit of excellence. However, the data collected in this study testify to the experienced difficulty of Olympic aspirants in determining foreseeable goals due to the reigning uncertainties associated with the pandemic.

#### Concerns About Future Performance

The novel situation of not being able to plan ahead, to have a set competition schedule to follow, to prepare for selected main events, and to aim for specific performance goals along the way seemed to increase concerns about building up one’s performance until the Olympic Games. This athlete summed it up: “High performance athletes are used to working toward their goals and to have a detailed schedule in mind. This one doesn’t exist in the current situation” (id4). As a consequence, “there is also an uncertainty, as I do not know how and where to train next year” (id5). The difficulty of staying competitive without opportunities to compare themselves to others, was described as a pressing issue by several respondents. Some participants experienced concerns about sustaining their form until the Olympics: “The already relatively high level has to be sustained for a year?!?” (id19), one participant seemed to ask in disbelief.

The uncertainty surrounding the ongoing Olympic qualification and impending cancelation of future qualification events was a recurring aspect in the data: “I was afraid that there would be no more competitions due to the corona crisis, which would destroy any possibility for me to reach the limits for a successful qualification” (id27). With the entire world in flux, Olympic athletes and coaches were faced with an unknown unpredictability of events.

Most notably, the concern of being subjected to more severe restrictions than their fellow competitors was noticeable across participants’ texts. “Many athletes do not have the possibility to train and they have to sit at home. Other nations, like Sweden, do not have such harsh restrictions for their population, and therefore don’t affect everyday life of athletes” (id23). When the pandemic caused a global standstill, athletes were stopped dead in their tracks to prepare for Olympic glory. Restrictions and mandates calling for physical distancing put in place made it impossible for them to continue their regular training as most facilities were closed. Still, many athletes tried to uphold their training routines in order to stay in shape and sustain their athletic capacity. Through social media, athletes and coaches in Austria found out that competitors in other parts of the world were still able to train in nearly normal formats. The feeling of being disadvantaged in comparison to fellow competitors could have acted as additional stressor, especially in the lock down phase. Overall, the impression was gained that various participants experienced the task of preparing for a peak in performance at the Olympic Games in the times of a global health crisis as a major challenge (cf. Schinke et al., 2020a).

#### Concerns About Living and Occupational Career

Several athletes and coaches expressed concerns about their future occupational career, and feared negative consequences for their daily living, most notably on a financial level due to the Olympic postponement. For example, one athlete stated: “Personally, the postponement implies a very high effort in organizational terms as well as in financial terms” (id25). These financial concerns were also emphasized by coaches: “Additionally it causes uncertainty: Will the athletes and myself receive the same funding? How will the sponsors react?” (id16). It became obvious that the postponement affected respondents’ scheduled plans for the time after the Olympic Games: “The decision what will happen after the Games will be postponed for another year. The plans which already existed for the time after the Games will also be postponed for a year” (id19).

As already shown in the last category, Olympic athletes’ and coaches’ statements revealed numerous concerns extending to multiple domains of their life. The statements might emphasize the assumption of increased stress levels in athletes and coaches as a consequence of diverse concerns and worries not only about the actual situation, but also their future perspective (Rajkumar, 2020; Schinke et al., 2020a,b). Since elevated stress levels have already been found in other subpopulations (Chakraborty, 2020; Mazza et al., 2020) as well as in elite athletes (di Fronso et al., 2020) as a psychological effect of COVID-19, the text data might hint at increased stress levels among these Olympic athletes and coaches, thereby supporting previous results. According to the transactional theory of stress by Lazarus and Folkman (1984), individuals can experience stress when the demands they are faced with exceed their perceived resources, endanger their well-being, and when making appraisals of threat, harm or challenge. As the participants in this study were confronted with an unprecedented situation, which was associated by many with negative effects in multiple domains of their life (e.g., sport, social life), the challenges naturally seemed difficult to meet. Consequently, higher levels of stress might be a negative psychological impact of the Olympic postponement among Olympic aspirants. Extant literature shows that performers in sport commonly experience organizational stress such as poor financial support structures, harsh selection criteria, or prolonged traveling time (Fletcher et al., 2012). In light of the knowledge that organizational stress can be associated with various problems for athletes (e.g., negative emotions, dissatisfaction, overtraining, poor psychological health, low well-being, burnout, and underperformance; Gould et al., 1999; Noblet et al., 2003; Meehan et al., 2004; Fletcher et al., 2012; Tabei et al., 2012), future research is needed to gain a more thorough understanding of the multiple stressors at play throughout the COVID-19 pandemic across elite athletes and coaches’ samples.

#### Opportunity of Performance Enhancement

The feature of having extra time at one’s disposal was a reoccurring topic in the data and can be interpreted in various ways. Multiple participants regarded the additional year as an opportunity to heighten their chances to compete at their best at the Olympics: “We are able to use this year to work on weaknesses and invest without pressure in things for which there would probably not be enough time under normal circumstances. You always have to see the positive I think” (id32). As this quote illustrates, many respondents intended to use the COVID-19 induced extra year to systematically work on personal weaknesses and described it as a chance for performance enhancement. Likewise, this athlete stated enthusiastically: “Would I have liked the Games to happen this year? Yes, of course, but on the other hand I am almost happier that I will be able to show even more what I am capable of next year. I can use the extra year to continue to train and to work on my weaknesses. All in all, I think that I would have made the Olympics this year and then I would have taken part in Tokyo. But next year, I will not only take part in Tokyo, but also show what I want to show!” (id27). Other participants shared this view by explaining how the structural changes made to their training environment in the previous year could now be parlayed into improved training conditions. Some athletes and coaches reasoned that the young age of the athletes turned the prolonged preparation phase until the Olympics into a relative advantage for them, since the additional time allowed them to close the gap to the leaders in their respective sport, and to develop their potential even further. Interestingly, helping athletes search for gaps and by this adopting a constructive problem-solving attitude, was recently suggested as an effective way of providing psychological support for athletes at the current stage of the pandemic by an international expert group of mental performance consultants (Schinke et al., 2020a).

#### Opportunity of Recovery

In high-performance sports, physical and psychological demands are experienced as a regular, yet challenging part of daily life. An important result of this study was therefore to see several athletes experience the postponement as an opportunity to recover from daily strains. One athlete asserted: “In my case the postponement of the Olympics reduced the pressure massively. For the first time in years, I have the time to process the events and emotions of the past time. … Now, for me personally, it is important to use the time wisely. Above all, to recover well, not only mentally but also physically” (id4). Another athlete also seemed glad to be afforded extra time to recover and additionally intended to use it to improve his/her performance in light of the ongoing selection: “Since I had to deal with injuries lately, the postponement of Tokyo 2020 gives me the time to prepare myself physically so far that I can qualify in the tournaments next spring” (id8). This view was shared by a participant who even predicted to be able to extend his/her athletic career by “another year or so” (id55) thanks to the possibility to heal the body in the gained off-season time.

All in all, the reflection that an extra year meant a personal advantage in comparison to others, was a common feature that was found in the data. Reading through the entirety of statements, the impression was gained that thanks to the unexpected pause and changed conditions, many participants finally had “time to breathe” in the fast-paced world of Olympic sport. A finding that emphasizes once more the amount of pressure that high-performance protagonists experience under normal circumstances.

### Coping With the Situation

Table 3 presents the codes, subcategories, and categories that were identified in participants’ texts relating to the theme of “Coping with the situation”. The dimensions were combined in the categories “Distancing from sport,” “Cognitive Reframing,” “Appealing for acceptance,” and “Planning behavior.” Since the distinguished dimensions are interconnected and overlap to some extent, the topics discussed as positive consequences of the Olympic postponement could also be interpreted as coping strategies that participants reverted to in order to adaptively respond to the situation imposed on them. Additionally, a single coping strategy could be sorted into more than one dimension (Compas et al., 1996).

#### Distancing From Sport

The differentiation between an initial, somewhat genuine emotional reaction and a later, more reflected and controlled response to the news of the delayed Olympic Games was noticeable in some of the quotes presented above. In fact, many respondents seemed happy, relieved and even thankful that they were afforded time to pursue other interests beyond sport and enjoy different aspects of life as a consequence of the Olympic postponement. This athlete expressed feeling closer to nature and enjoying training outside, a welcome change for an indoor athlete. “At this time of year, I have never before felt sunrays on my face this often as at the moment” (id4). Another athlete enjoyed having more time to spend with his/her children, a wonderful side-effect of the sudden changes in season planning. While competing and training abroad may represent an attractive way of life for many, ceased travel obligations were mentioned as a positive impact of the Games’ postponement in this study. As this athlete suggested: “Personally, I find it enjoyable as a whole, for the first time, a time without travel, stress and pressure” (id30). This athlete concludes by asserting that this extra time allowed them to finish off other tasks such as completing their master thesis and to thereby free their head.

All these comments show that athletes also reflected on topics beyond training and performance. As diverse uncertainties were mentioned by athletes and coaches relating to the postponement of the Games, exploring interests other than sport might serve as a coping strategy to deal with these potential stressors (Nicholls and Polman, 2007). Athletes and coaches distanced themselves from sport and turned their attention away from the incisive decision and its ramifications to more positive and/or controllable aspects of their daily lives. Being able to open up to other life domains and personal interests beyond sport such as focusing on nature experience, leisure time and hobbies, family, friends and/or university studies, might serve as distraction for these Olympic aspirants. Following this interpretation, the attempt to distract oneself can be seen as an avoidance coping strategy (Folkman and Moskowitz, 2004; Polman, 2011). Individuals use avoidance coping primarily in situations in which they perceive themselves to have little control over the outcome (Carver et al., 1989; Nicholls and Polman, 2007), such as when goals are unreachable irrespective of the effort made. In such situations, the strategy of avoidance seems to be an effective way of coping with acute stressors (e.g., not thinking about a faulty referee decision), whereas in the long-term negative consequences such as increased negative affectivity (Ingledew et al., 1997), more pronounced symptoms of burnout (Polman et al., 2010), and higher levels of stress (Polman, 2011) were reported. While providing short-term relief of negative emotional states and inner experiences, avoidance coping in principle indicates that an individual is turning away from a problem, thereby deciding not to confront it. Yet, if an individual cannot physically or mentally disengage from a situation that he/she does not want to be exposed to, such as the current global health crisis encompassing every life domain, it is likely that he/she will experience increased stress as a consequence of trying to escape the situation in vain (Carver and Scheier, 1998; Folkman and Moskowitz, 2004).

An alternative way of interpreting the statements made by the study participants is the idea of broadening their athletic identity. By allowing themselves to explore a breadth of interests, athletes and coaches could use this historical time to grow in a holistic sense by developing their personal multifaceted identity. As a unidimensional athletic identity has been identified as a risk factor for developing symptoms of burnout (Coakley, 1992; Gustafsson et al., 2008, 2018), promoting a broader identity development in their clients and following a *whole person approach* could be an important intervention for practitioners working with Olympic athletes and coaches in these challenging times (Wylleman et al., 2013; Schinke et al., 2020b; Stambulova et al., 2020).

#### Cognitive Reframing

Several of the consequences of the Olympic postponement perceived as personally advantageous by participants, such as a relative age advantage over other competitors, using the additional time to optimize training and improve performance, regarding the situation as a unique opportunity to recover physically and mentally, and to explore other interests, can all be classified as positive reframing of the situation. Likewise, the statement, “but a postponement is still better than a complete cancelation, which would have meant having wasted years of training and fighting” (id25), can be understood as reframing of the situation, which consequently fosters acceptance of the postponement, an event that cannot be changed or controlled itself. This cognitive strategy is a psychological technique that helps a person to view things differently by putting them in a different context, thereby allowing new perspectives to arise (Robson and Troutman-Jordan, 2014). As such, it refers to a general shift in a person’s mindset. In this regard, athletes and coaches might apply meaning-focused coping strategies, as they re-evaluate the positive meaning and outcomes of the postponement, thereby engaging in benefit-finding (Tennen and Affleck, 2002). Past research already emphasized successful adaptations, especially in less controllable situations, through meaning-focused coping attempts (Folkman and Moskowitz, 2004; Guo et al., 2013).

Additionally, different lines of arguments were presented why the postponement was the only viable option under the exceptional and unique circumstances, such as difficult and scarce training conditions for athletes world-wide in the preceding weeks. As mentioned earlier, the training restrictions varying to a great extent locally and internationally received a lot of interest, as visible in this athlete’s quote: “Moreover, training conditions were very different from country to country, the postponement of the Games allows for a fair preparation” (id17). The idea of fairness was expressed by many participants, as it represents one of the core Olympic values and is translated in the notion of a level playing field for all competitors. One athlete said: “I definitely think the postponement is the right solution to ensure equality of opportunity for all athletes around the world” (id27). Another prominent feature along the lines of having the bigger picture in mind when judging the IOC’s decision, was the plea for regarding health in general as the most important asset and therefore treating it as foremost priority: “From a health perspective, it was certainly the right decision to postpone the Games. The risk would have simply been to big internationally” (id12). One participant added a personal experience: “Health is the most important issue. This became increasingly clear to me as an athlete during the course of my career and was confirmed one more time now” (id13).

In these statements, it seems that athletes and coaches attempted to view their actual situation and its personally experienced implications from a broader perspective. In fact, in many cases they tried to legitimize their worries and doubts by opposing their situation to others. The comparison to fellow athletes and coaches, who potentially face more severe restrictions and consequences, could help to judge the actual situation as less dramatic and thus, to feel less disadvantaged. In case the depicted attitude reflects their genuine beliefs, these participants seemed to have found effective ways of coping already, which might display these athletes’ and coaches’ high level of expertise (cf. Nicholls et al., 2007).

#### Appeal for Acceptance

Participants also presented personal maxims that seemed to help them respond with poise to the unprecedented challenges imposed on them. As this athlete explained: “Anyway, I accept this decision and it can always be positive for one person and negative for another. For me, the most important is the realization that everything shall happen as it may (that is my personal way of handling the situation) and to try and make the best of the situation” (id4). Another participant declared living the situation by the principle of “One has to accept the situation as it is” (id38). The last two quotes suggest that participants employed these philosophies or mottos with a motivational and emotional purpose. As such, they can be interpreted as an attempt to adopt a mindful attitude of non-judgmental awareness to what really is instead of ruminating about what was and what could be. Several mindfulness approaches specifically targeted at high-performance athletes and coaches demonstrate that such a poised attitude based on the (trained) ability to make room for uncomfortable internal and external experiences (e.g., thoughts, feelings, or a sudden pandemic ruining all plans for the next future), while still engaging in personally valued activities, could allow athletes and coaches to navigate the broader challenges of a high-performance environment in a mentally and physically healthy way (Baltzell and Summers, 2017; Kaufman et al., 2018). Such a feeling of acceptance also represents an essential requirement for meaning-focused coping to enable personal growth in personally demanding or rather overburdened situations (Folkman and Moskowitz, 2004; Guo et al., 2013).

Yet, in some statements, a very strong appeal for acceptance and an almost forced optimism in handling the situation positively, became apparent. In particular, one coach wrote: “The postponement is necessary and because of that you have to accept it” (id41). Another coach stated: “In no way can this allow the motivation to drop… You always have to see the positive things, I think” (id16). Consciousness about an athlete’s societal duties and thus assuming this responsibility by acting as a good example in taking an optimistic stance and behaving accordingly, was another feature that became apparent in this athlete’s text: “I am firmly convinced that we (as) athletes have a duty toward society – namely as role models to approach the future positively” (id22). These statements suggest that athletes and coaches made conscious efforts to accept the postponement, evoking the image of an inner dialog, in which a statement is continuously repeated like an incantation in order to convince oneself at the end by sheer force of repetition.

However, the interpretative nature of the conducted content analysis in this study makes it possible for the derived features to be viewed in differing ways. Hence, the fact that participants when being invited to express their subjective views and describe their very personal experiences, responded by presenting ethical arguments and life mottos, could also be regarded as serving different functions. First, it could be an attempt to portray oneself in a socially desired way, and by expressing sympathy for the necessary restrictions and ramifications of the COVID-19 pandemic for the entire world, participants took a moral stance as general members of society. Second, being bound by commitments toward various institutions, stakeholders and sponsors in the world of sport, participants felt obliged to act as athletic role models for society, implying self-presentation in favorable ways, and the display of outstanding character strengths in the face of adversity. Adding to this interpretation, participants mentioned Olympic values such as fairness in order to demonstrate how they were worthy members of the Olympic family, well aware of non-debatable principles. Third, study participants did not trust the anonymous setting that was explained extensively to them in the lead-up to the open-format questions. As mentioned earlier, high-performance sport in Austria is a small universe in which protagonists know each other personally in most cases. It is therefore possible that participants wanted to ensure that their answers contained moral arguments in case their identities would be revealed. It can also be hypothesized that the disbelief in anonymity is based on a general ignorance of (or even mistrust in) scientific standards. In this sense, the setting would have been perceived not much different to that of a media interview in which self-presentation is of upmost importance.

#### Planning Behavior

Another possibility to cope with the situation seemed to be achieved through planning behavior. Several athletes and coaches discussed scenarios of how to benefit from the postponement of the Olympic Games. It seemed that generating options for a more beneficial progress of athletic performance was a common strategy to handle the postponement of the Olympic Games. For example, one athlete stated: “Above all, the focus is on a long build-up training cycle, which hopefully prepares us well for the upcoming competition season” (id4) or rather “concerning training I am already in a basic preparation phase again. Besides endurance and torso training I try to evaluate my technique and bring it to perfection point by point” (id22). Also, from the coaches’ perspective, planning behavior as a strategy to cope with the situation was applied: “From this time on, we had already worked on different scenarios to enable corresponding adaptions in athletes training” (id41). By making detailed plans, coming up with concrete training programs to follow and performance goals to strive for, athletes and coaches were able to take ownership of their (athletic) life again, thereby regaining more control over their daily structure. The prospect of a more predictable future might help participants live the time of the Olympic standstill in an adaptive way. Thus, planning behavior could be interpreted as a problem-focused coping strategy, as it consists of taking actions to change the person-environment relationship (Lazarus, 1999).

Collectively, the data demonstrated that study participants employed different psychological strategies to deal with the situational demands they were facing as a consequence of the Olympic postponement. Despite a longstanding tradition of coping research, there is lack of agreement regarding adaptive forms of coping in sport until this day (for a systematic review see Nicholls and Polman, 2007; Polman, 2011). According to Nicholls (2010b), coping effectiveness is the “degree in which a coping strategy or combination of strategies is or are successful in alleviating stress” (p. 264). Following this understanding, only few studies have looked into the issue of coping effectiveness of strategies used by elite athletes and coaches in high-performance sport, producing inconsistent evidence in addition (Polman, 2011). The findings of this study underline the importance for future research to delve into this area of interest in order to advance a more thorough understanding of effective coping in elite sport. In particular, sport psychologists and mental consultants need to gain scientifically derived knowledge to be able to design and implement advisable coping strategies for their clients (Folkman and Moskowitz, 2004), especially in challenging times like the current pandemic.

### Limitations

The dimension of extra time was not conceptualized as a theme on its own in this study. Nevertheless, throughout the analyses the impression was gained that the issue of time itself translated into having to wait for another year, represented a major challenge. An athlete wrote about a lengthy Olympic preparation phase of over 10 years, and his/her statement evolved from a seemingly objective description of the current situation to an increasingly reflexive monolog. This transition is only discernable when analyzing this participant’s statement as a whole. It can be addressed as limitation of this study that data analysis was done by sequence through the examination of meaning units, so that the evolvement of participants’ statements could not be detected. In order to allow for more in-depth analysis and with the goal to glean an even deeper understanding of the psychological experience, the authors suggest choosing entire accounts as unit of analysis for future studies. Another important limitation was the fact that original quotes had to be translated from German to English by the authors. Since the study was of interpretative nature, changing text data might interfere with the derivation of meaning. Yet another limitation, which can be considered an ethical dilemma, is related to the disbelief of study participants in the anonymity of the setting. If this is due to the presumed interconnectedness within Austrian high-performance sport or based on lacking knowledge regarding scientific principles, can only be hypothesized.

## Conclusion

This qualitative study was aimed at exploring participants’ subjective perceptions in order to glean insights into how Olympic athletes and coaches experience the postponement of the Olympic Games in Tokyo 2020. The findings derived from an exclusive sample of aspiring Olympic athletes and coaches from Austria, and data collection was conducted at the beginning of the COVID-19 lockdown, when predictions for its duration were vague. The scope of transferability of the reported findings might therefore be limited. However, qualitative research in general is aimed at obtaining rich and contextually sensitive data, thereby producing local knowledge (Marecek, 2003), so that the contextual embeddedness is not a limitation but the main characteristic of this study.

The use of short written statements in a web-based format as a data collection strategy can be considered as a contribution to qualitative research methods within sport and exercise psychology. When planning the research design, the authors expected participants to be inundated by the constant influx of digital information, and thus be unwilling to spend additional time online to support the study’s scientific interest. It seemed, however, that study participants used the opportunity to process their emotions and thoughts through verbalizing their experiences. Effects of expressive writing on well-being have been studied since Pennebaker’s and Beall’s seminal study (Pennebaker and Beall, 1986) in psychological research. In this line, putting personal experiences in words in a web-based format allows participants to perpetuate, test, delete, reorganize and reformulate and thereby process their experiences directly (Schiek, 2014).

The variety of topics that were identified in the data analyses was an important result and supports the authors’ constructivist beliefs that each participant is unique in his/her engagement with the Olympic postponement and its implications. The findings are also in line with the assumptions made from a holistic development perspective (Wylleman, 2019), namely the idea that athletes face changes on multiple levels, such as in their athletic but also academic-vocational development due to the postponement. While some topics (e.g., concerns about general health and fairness) were consistent with arguments found in media interviews and social media comments of athletes and coaches, others came as a surprise and showed how the situation was experienced like an emotional rollercoaster by some participants. As such, the finding that athletes even considered a premature retirement to escape the overwhelming physical and mental requirements associated with the undue year of preparation was quite significant. The feeling of exceeding demands in the pressurized high-performance environment of Olympic sports was clearly discernable in the data.

On the other hand, the finding that many athletes and coaches alike viewed the postponement as a chance for pursuing complementary interests beyond sport, improvement and recovery was a promising result, demonstrating their ability to cope with adversity. It also hints at the fact that the involuntary pause yields holistic opportunities for growth (Schinke et al., 2020b). As a consequence, in providing mental services to athletes and coaches during the ongoing pandemic, mental performance consultants should take a reflexive stance and go about this task in a mindful (i.e., non-judgmental and curious) way in order to appreciate each individual’s perspective and idiosyncrasy in this historical time. For example, the finding that some athletes benefited from the pause by taking care of physical issues (such as long-term injuries) and mental aspects (such as emotional exhaustion) could inform the direct work of high-performance coaches as well as sport psychologists by educating them about the fact that not every athlete needs new goals to strive for or severe training regimes to follow in this unprecedented situation. On the contrary, drawing on the authors’ direct experience in working with Olympic aspirant coaches and athletes in recent months, some athletes might seize the opportunity to breathe and enjoy living in a pressure-free space in the lead-up to the Olympic Games. Assisting athletes in self-reflecting and thereby allowing them to accept their very personal interpretation of the current situation can be an important task for mental consultants as well as coaches, which can be derived through this study.

The interpretative focus of the data analyses holds that there may be more than one valid and useful set of findings from a given data set (Levitt et al., 2018). Hence, the first author emphasizes the understanding that the mentioning of rational arguments to support the postponement along with motivational mottos is due to the constraints, which the roles of Olympic athletes and coaches entail. As discussed earlier, their answers reflect how they expect people would want to see them (i.e., their perceived public image), are employed quasi strategically, and can be interpreted as side-effects of being socialized in a high-performance environment. This understanding of findings is done from the position of being a retired Olympic elite athlete who experienced the aforementioned constraints and expectations herself over the course of nearly two decades. By acknowledging this personal perspective, the first author assumes a reflexive stance and exemplifies that the production of theory-free knowledge is impossible.

This article intends to inform the development of evidence-based interventions that can be utilized to alleviate associated negative effects and promote Olympic aspirants’ well-being during insecure times like the ongoing COVID-19 pandemic. One possible implication for future practice of mental performance consultants is to employ strategies that foster openness in terms of the broad array of feelings and thoughts that Olympic aspirants are experiencing and support them in developing an agile mindset (Schinke et al., 2020a). Adopting an attitude characterized by psychological flexibility allows for such a mindset and could be best suited to help these top performers cope with the looming cancelation of the Tokyo 2020 Games and potential negative consequences thereof. Mindfulness approaches for performance sport contexts aim at promoting psychological flexibility (e.g., Gardner and Moore, 2007) and could be one specific method to assist athletes and coaches in these challenging times. For the near future, additional data on how other international groups of aspiring Olympic athletes and coaches experience the historical postponement and its implications is desirable. For example, through longitudinal monitoring of how this population continue to respond to the postponement and rescheduling of the 2020 Olympic Games, case studies of athletes and coaches who aim to compete at the rescheduled Games, and tracking athletes’ progress through specific phases and/or over long-term periods until the conclusion of this major competition. Derived insights could ultimately facilitate the development of effective psychological approaches to support Olympic athletes and coaches in their personal endeavor in sport and life in general.

## Data Availability Statement

The raw data supporting the conclusions of this article will be made available by the authors, without undue reservation.

## Ethics Statement

Ethical review and approval was not required for the study on human participants in accordance with the local legislation and institutional requirements. The patients/participants provided their written informed consent to participate in this study.

## Author Contributions

Both authors conceived and designed the study, collected the data, conducted the data analyses and contributed to writing the manuscript.

## Conflict of Interest

The authors declare that the research was conducted in the absence of any commercial or financial relationships that could be construed as a potential conflict of interest.
